# Introduction and transmission of SARS-CoV-2 lineage B.1.1.7, Alpha variant, in Denmark

**DOI:** 10.1186/s13073-022-01045-7

**Published:** 2022-05-04

**Authors:** Thomas Y. Michaelsen, Marc Bennedbæk, Lasse E. Christiansen, Mia S. F. Jørgensen, Camilla H. Møller, Emil A. Sørensen, Simon Knutsson, Jakob Brandt, Thomas B. N. Jensen, Clarisse Chiche-Lapierre, Emilio F. Collados, Trine Sørensen, Celine Petersen, Vang Le-Quy, Mantas Sereika, Frederik T. Hansen, Morten Rasmussen, Jannik Fonager, Søren M. Karst, Rasmus L. Marvig, Marc Stegger, Raphael N. Sieber, Robert Skov, Rebecca Legarth, Tyra G. Krause, Anders Fomsgaard, The Danish COVID-19 Genome Consortium (DCGC), Kasper S. Andersen, Martin H. Andersen, Amalie Berg, Susanne R. Bielidt, Sebastian M. Dall, Erika Dvarionaite, Susan H. Hansen, Vibeke R. Jørgensen, Rasmus H. Kirkegaard, Wagma Saei, Trine B. Nicolajsen, Stine K. Østergaard, Rasmus F. Brøndum, Martin Bøgsted, Katja Hose, Tomer Sagi, Miroslaw Pakanec, David Fuglsang-Damgaard, Mette Mølvadgaard, Henrik Krarup, Christina W. Svarrer, Mette T. Christiansen, Anna C. Ingham, Thor B. Johannesen, Martín Basterrechea, Berit Lilje, Kirsten Ellegaard, Povilas Matusevicius, Lars B. Christoffersen, Man-Hung E. Tang, Kim L. Ng, Sofie M. Edslev, Sharmin Baig, Ole H. Larsen, Kristian A. Skipper, Søren Vang, Kurt J. Handberg, Marc T. K. Nielsen, Carl M. Kobel, Camilla Andersen, Irene H. Tarpgaard, Svend Ellermann-Eriksen, José A. S. Castruita, Uffe V. Schneider, Nana G. Jacobsen, Christian Ø. Andersen, Martin S. Pedersen, Kristian Schønning, Nikolai Kirkby, Lene Nielsen, Line L. Nilsson, Martin B. Friis, Thomas Sundelin, Thomas A. Hansen, Marianne N. Skov, Thomas V. Sydenham, Xiaohui C. Nielsen, Christian H. Schouw, Anders Jensen, Ea S. Marmolin, John E. Coia, Dorte T. Andersen, Mads Albertsen

**Affiliations:** 1 Department of Chemistry and Bioscience, Aalborg University, Aalborg, Denmark; 2 Centre of Excellence for Health, Immunity and Infection (CHIP), Department of Infectious Diseases, Rigshospitalet, University of Copenhagen, Copenhagen, Denmark; 3 Department of Applied Mathematics and Computer Science, Technical University of Denmark, Lyngby, Denmark; 4 Infectious Disease Epidemiology & Prevention, Statens Serum Institut, Copenhagen, Denmark; 5 Infectious Disease Preparedness, Statens Serum Institut, Copenhagen, Denmark; 6 Unit for Research Data Services (CLAAUDIA), Aalborg University, Aalborg, Denmark; 7 Department of Virus & Microbiological Special Diagnostics, Statens Serum Institut, Copenhagen, Denmark; 8 Center for Genomic Medicine, Rigshospitalet, Copenhagen, Denmark; 9 Department of Bacteria, Parasites and Fungi, Statens Serum Institut, Copenhagen, Denmark; 10 https://www.covid19genomics.dk/about

## Abstract

**Background:**

In early 2021, the SARS-CoV-2 lineage B.1.1.7 (Alpha variant) became dominant across large parts of the world. In Denmark, comprehensive and real-time test, contact-tracing, and sequencing efforts were applied to sustain epidemic control. Here, we use these data to investigate the transmissibility, introduction, and onward transmission of B.1.1.7 in Denmark.

**Methods:**

We analyzed a comprehensive set of 60,178 SARS-CoV-2 genomes generated from high-throughput sequencing by the Danish COVID-19 Genome Consortium, representing 34% of all positive cases in the period 14 November 2020 to 7 February 2021. We calculated the transmissibility of B.1.1.7 relative to other lineages using Poisson regression. Including all 1976 high-quality B.1.1.7 genomes collected in the study period, we constructed a time-scaled phylogeny, which was coupled with detailed travel history and register data to outline the introduction and onward transmission of B.1.1.7 in Denmark.

**Results:**

In a period with unchanged restrictions, we estimated an increased B.1.1.7 transmissibility of 58% (95% CI: [56%, 60%]) relative to other lineages. Epidemiological and phylogenetic analyses revealed that 37% of B.1.1.7 cases were related to the initial introduction in November 2020. The relative number of cases directly linked to introductions varied between 10 and 50% throughout the study period.

**Conclusions:**

Our findings corroborate early estimates of increased transmissibility of B.1.1.7. Both substantial early expansion when B.1.1.7 was still unmonitored and continuous foreign introductions contributed considerably to case numbers. Finally, our study highlights the benefit of balanced travel restrictions and self-isolation procedures coupled with comprehensive surveillance efforts, to sustain epidemic control in the face of emerging variants.

**Supplementary Information:**

The online version contains supplementary material available at 10.1186/s13073-022-01045-7.

## Background

A year into the SARS-CoV-2 pandemic, it became clear that variants with increased transmissibility and/or reduced vaccine efficacy would have a profound impact on our lives, including prolongation of the pandemic as well as increasing morbidity and mortality. On 14 December 2020, Denmark received a notification from the Early Warning and Response System of the European Union (EWRS) about a novel SARS-CoV-2 lineage (B.1.1.7 [1]) spreading fast throughout the United Kingdom (UK). The notification was a direct response to genomic sequences uploaded to the GISAID [2] database by the Danish COVID-19 Genome Consortium (DCGC). On 18 December 2020, the New and Emerging Respiratory Virus Threats Advisory Group (NERVTAG), advisers to the British Health Authorities, released a public statement, reporting a 71% (95% CI: [67%, 75%]) increased transmissibility for the B.1.1.7 lineage and elevated it to variant-of-concern (VOC) [3]. The B.1.1.7 lineage was first detected in the UK in September 2020 and has since spread across the world [4], including Denmark where the first identified case was sampled on 14 November 2020 [5]. Several studies have since estimated an increased transmissibility of B.1.1.7, ranging from 29 to 130% [6–9]. This is in line with early estimates of 36%-55% from Denmark [10, 11]. Hypotheses for increased transmissibility include higher viral load, longer infection period, and better receptor binding [1, 12, 13].

The B.1.1.7 lineage contains several genetic changes compared to the specimens first identified in Wuhan, China. Most notably is the spike protein amino acid substitution N501Y which enhances binding to the ACE2 receptor and is thought to increase transmissibility [1]. Acquired mutations can be used to construct the phylogenetic relationship between genomes and by coupling with temporal information, transmission timing and dynamics of emerging lineages can be inferred [14]. These approaches have been used by several studies to gain insights into the SARS-CoV-2 pandemic on a national level [8, 9, 15–19].

Due to the early warning from the UK and an already extensive sequencing effort in Denmark, it was possible to predict growth dynamics for B.1.1.7 in Denmark, when it was still less than 2% of all circulating SARS-CoV-2 lineages. Hence, on 2 January 2021, B.1.1.7 was predicted to exceed 50% relative abundance nationwide in the middle of February [5, 20], which formed a key argument in the political decision to further extend the lockdown at the time and intensify contact tracing on all B.1.1.7 cases [21]. In this study, we utilize data from 175,213 SARS-CoV-2 positive cases, to substantiate early results by providing an updated estimate of increased transmissibility of B.1.1.7. Furthermore, using genome isolates from 1976 case of B.1.1.7 with detailed and comprehensive metadata, we outline the dynamics of B.1.1.7 in Denmark to evaluate the impact of introductions and regional restrictions on the prevalence and spread of B.1.1.7.

## Methods

### Study data

**Fig. 1:**
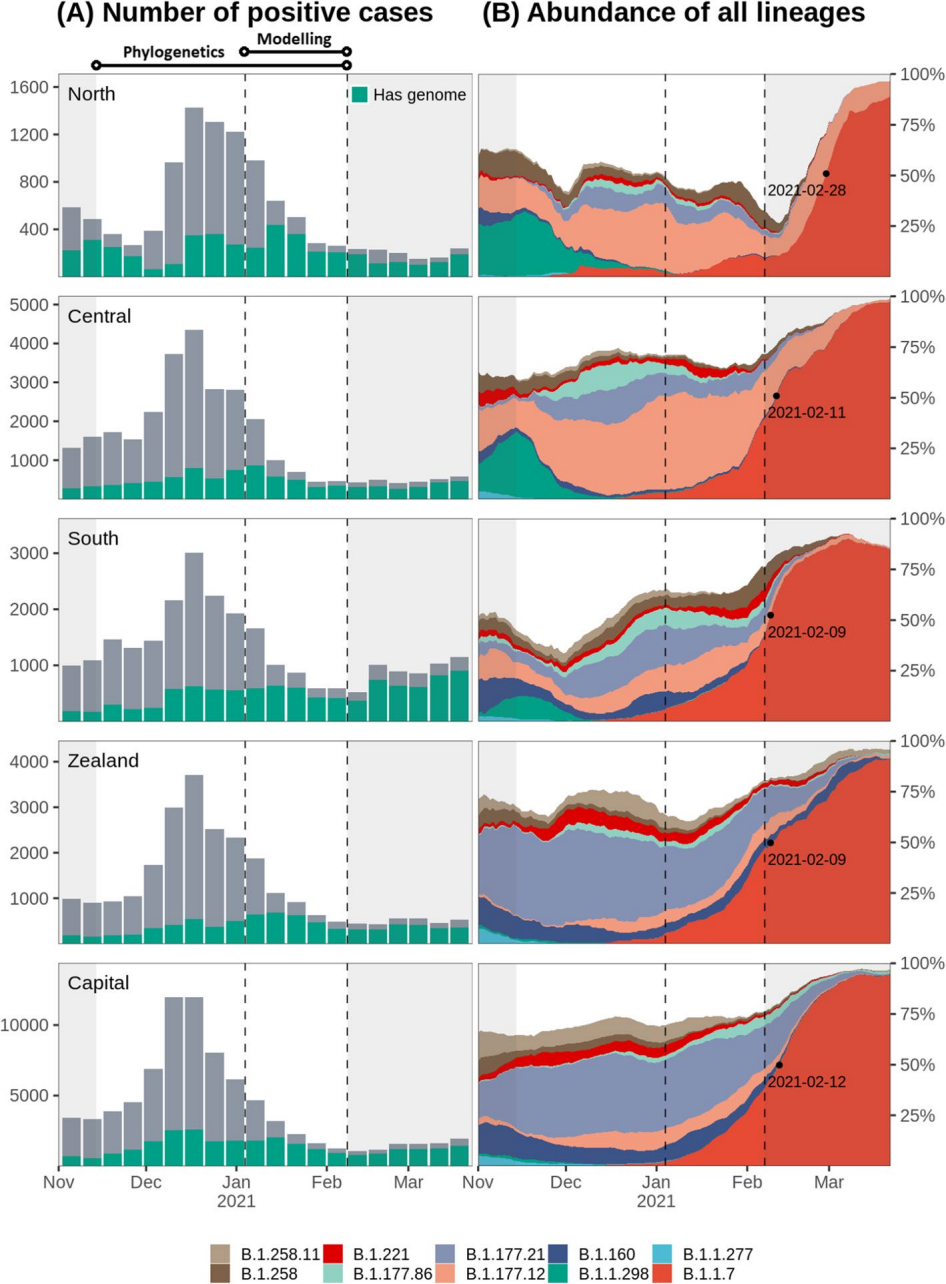
Sequencing rate and lineage dynamics. Each row corresponds to the region highlighted to the far left. The two vertical dashed lines indicate the beginning and end of study period used to infer B.1.1.7 transmissibility, while the non-shaded area shows the period used for phylogenetic analysis. The time outside the study are shaded in gray. **A** Sequencing rate across time. Total height corresponds to the total number of cases for each week. Green bars indicate the number of cases for which a high-quality genome was available (less than 3000 ambiguous bases). **B** Relative abundance of ten most abundant PANGO lineages across Denmark in the study period. Computed from rolling averages of daily cases with genome available over a 14-days window. Less abundant lineages are not shown but included in the unfilled area of the plots. The black dot and date corresponds to the point where B.1.1.7 crosses 50% relative abundance

We used comprehensive register data obtained from Statens Serum Institute (https://www.ssi.dk/) from all 175,213 persons with a positive SARS-CoV-2 reverse transcription polymerase chain reaction (RT-PCR) test in Denmark, within the study period. Detailed epidemiological records were retrieved from the Danish Patient Safety Authority [22]. We used the Danish civil registration number, which is a unique personal identifier, to link the two datasets. Daily numbers of RT-PCR tested individuals were obtained from https://covid19.ssi.dk [23]. We restricted the study period to include the first observation of B.1.1.7 in Denmark on 14 November 2020 (week 46) until 7 February 2021 (week 5) which marked the reopening of Danish primary schools. To estimate transmissibility, we focused on a period with stable restrictions from 4 January (week 1) to 7 February 2021 (week 5). Personal official addresses were used to define the geographical position of each case, which was aggregated to the five regions of Denmark (North, Central, South, Zealand, and Capital. See Fig. 1C for geographical overview).

### Sequencing data

Throughout the study period, a subset of 77,074 positive RT-PCR tests were selected for sequencing by The Danish COVID-19 Genome Consortium (DCGC), established in March 2020 to assist public health authorities by providing rapid genomic monitoring of the spread of SARS-CoV-2. Selection was done by cycle threshold (Ct) values, using cutoffs between 30 and 38 in the study period [24]. Whole genome amplification of SARS-CoV-2 was performed using a modified version of the ARTIC tiled PCR scheme [25] targeting a total 33 overlapping amplicons between 1000 and 1500 bp, and a custom 2-step PCR strategy was used for barcoding the amplicon libraries. Barcoded libraries were normalized and pooled and prepared for sequencing with the SQK-LSK109 ligation kit (Oxford Nanopore), and sequencing was performed on the MinION device using R.9.4.1 flowcells (Oxford Nanopore). A complete protocol can be found at protocols.io [26]. The raw sequencing data was basecalled using Guppy v.3.6.1 (https://nanoporetech.com) and demultiplexed using a custom cutadapt v.2.10 [27] wrapper. Generation of consensus sequences were done using the function “artic minion” with default settings from the Arctic Network bioinformatics protocol v.1.1.0 [28], which uses Medaka v.1.0.3 (https://nanoporetech.github.io/medaka/) for consensus calling. The consensus sequences were masked in the beginning (54 bases) and end (67 bases) to avoid primer biases. A consensus sequence with less than 3000 ambiguous bases (approx. 10% of the genome) were considered a “high-quality genome” and used in the national SARS-CoV-2 surveillance. If not, the consensus sequence was considered failed and not used. A haplotype was assigned to a consensus sequence, if there existed > 1 consensus sequence with the same set of nucleotide mutations. Nucleotide mutations of designated haplotypes used in this study are listed in Additional file 1: Table S1.

### Calculating transmissibility

We modeled the daily counts of B.1.1.7 and all other variants using Poisson regression:

$${P}_t={I}_t{T}_t^{\beta }{S}_t$$

Here, *P*_*t*_ is the expected daily counts, *T*_*t*_ is the daily number of tested, and *S*_*t*_ is the proportion of positive tests with a genome. *I*_*t*_ is a measure of incidence depending on the location (Danish region), time (date), and lineage (either B.1.1.7 or any other lineage). *S*_*t*_ and *T*_*t*_ are given as input. As activity in society fluctuates systematically over time this leads to autocorrelation in the observed number of cases. We incorporated an AR1 autocorrelation structure of date for each region separately, using the glmmTMB R-package [29]. Using R notation this leads to the following formula specification:

$$Pt\sim Region+ Date+ Type+ Region: Type+ Date: Type+\mathit{\log}(Tt)+ offset\left(\mathit{\log}(St)\right)+ ar1\left( Date+0| Region\right)$$

where Type indicates either B.1.1.7 or all other lineages. There are interactions between Type and Region and Date to allow for different prevalence for the different types in the different regions and different growth rates, respectively. *β* is estimated by adding *log(T*_*t*_*)* and *log(S*_*t*_*)* is included as an offset—the logarithms are due to using log as the natural link function in the generalized linear model. We also explored alternative relevant models and compared them to our chosen model using the Akaike Information Criterion (AIC). As we observed a substantial difference in the development of B.1.1.7 in the North region, we tested if a region-specific estimate of growth rate could be justified by including the term *Region:days* to the model:

$$Pt\sim Region+ Date+ Type+ Region: Type+ Date: Type+ Region: days+\mathit{\log}(Tt)+ offset\left(\mathit{\log}(St)\right)+ ar1\left( Date+0| Region\right)$$

The AIC increased by 6 (2306.9 to 2312.9) and none of the region-specific growth rates were significant (all *p* > 0.25). We also verified that the autocorrelation structure was needed, by fitting a reduced model without the *ar1(Date + 0|Region)* term:

$$Pt\sim Region+ Date+ Type+ Region: Type+ Date: Type+\mathit{\log}(Tt)+ offset\left(\mathit{\log}(St)\right)$$

As AIC increased substantially by 112 (2306.9 to 2418.9), the autocorrelation structure was not dropped from the model.

### Phylogenetic analysis

Consensus sequences from DCGC were collected until 17 April 17, 2021, and global reference consensus sequences from GISAID [2] were downloaded 15 April 2021. These were labeled with phylogenetic clades and lineages with Pangolin v2.2.2 [30] using the PANGO nomenclature [31]. The data was subset to B.1.1.7 only. The global reference data was then downsampled at random to maximum 20 sequences per collection week and country. Sequences were aligned to the Wuhan-Hu-1 MN908947.3 reference using mafft v7.471 [32]. We then constructed an initial maximum likelihood tree using IQ-tree v2.0.3 using a general time reversible model [33] with empirical base frequencies and a free-rate model [34] including three categories. This model was predicted as the best model for maximum likelihood phylogenetic construction assessed using ModelFinder [35]. From the initial tree, sequences with > 3 times residuals from the interquartile range in a root-to-tip regression [36] and sequences with branch lengths longer than the 99% percentile of the branch length distribution were discarded. We generated a maximum likelihood tree from the filtered sequences using the model previously identified and ran 1000 ultrafast bootstrap (UFboot [37]), iterations to assess branch support.

To time-scale the maximum likelihood tree we used LSD2 v1.9.7 [38], with Wuhan-Hu-1 as an outgroup. We fixed the clock-rate to 5.6-e4 as reported for B.1.1.7 previously [1] and collapsed branches with lengths < 1e5 to reduce noise. The -e option was set to four, removing sequences that deviated more than 4 standard deviations from the branch length distribution. After quality filtering a total of 1976 Danish and 3611 international B.1.1.7 genomes remained.

The time-scaled tree was used as input to pastml v1.9.33 [39] which we ran with default settings except setting the *--resolve_polytomies* option, as polytomies can inflate the number of introductions in the analysis [40]. PastML was used to reconstruct ancestral states using a four state F81-like model for nucleotide substitution generalized to the number of geographic states [41]. Under the F81-like model, the migration rate from a state *i* (e.g. geographic region) to a different state *j* (*i ≠ j*) is proportional to the equilibrium frequency of *j*, termed *𝞹j*. The rescaling factor which is analogous to the mutation rate under a strict molecular clock is optimized in PastML, in addition to the state equilibrium frequencies. In other words, the rescaling factor represents the average number of character changes per year for time-scaled trees. This is applied to all tree branches, which represents the average number of character changes per branch unit. Setting the *–resolve_polytomies* option, geographic group labels are used to resolve polytomies and create subtrees for each unique group. This procedure takes place after ancestral inference has been performed [40].

## Results

A total of 175,213 SARS-CoV-2 positive cases were registered in Denmark in the period of this study between 14 November 2021 and 7 February 2021. Of these, 77,074 (44%) was sequenced with 60,178 (34%) yielding a high-quality genome. The genome coverage relative to the total number of cases was comparable between regions throughout the study period, with the exception of the North region during November 2021 (Additional file 1: Fig. 1A and S1A). The increased genomic surveillance and testing effort (Additional file 1: Fig. S1B) was due to the rapid expansion of the mink-related lineage B.1.298 [42], which was the most abundant lineage in North and Central regions at that time (Fig. 1B).

We modeled the growth rate of B.1.1.7 relative to other lineages in the period from 4 January to 7 February 2021. The study period was chosen as both the PCR testing and sequencing capacity increased substantially until January, reaching a weekly rate of > 13,000 tests per 100,000 people, a positive percent below 4%, and > 75% of all positive samples having a high-quality genome (Fig. 1A, Additional file 1: Fig. S1). Furthermore, a window with stable restrictions was enforced from January 4th until partial reopening of primary schools from February 8th. A Poisson regression model was fitted on daily counts of B.1.1.7 to estimate increased transmissibility of B.1.1.7 relative to all other lineages. We found a growth rate of 0.021 and − 0.062 per day for B.1.1.7 and other lineages respectively, corresponding to an increased transmissibility of 58% (95% CI: [56%, 60%]) for B.1.1.7 assuming a generation time of 5.5 days [12, 43]. We did not observe significant differences in transmissibility between regions (${\chi}_{(4)}^2=3.17,p=0.54$).

**Fig. 2:**
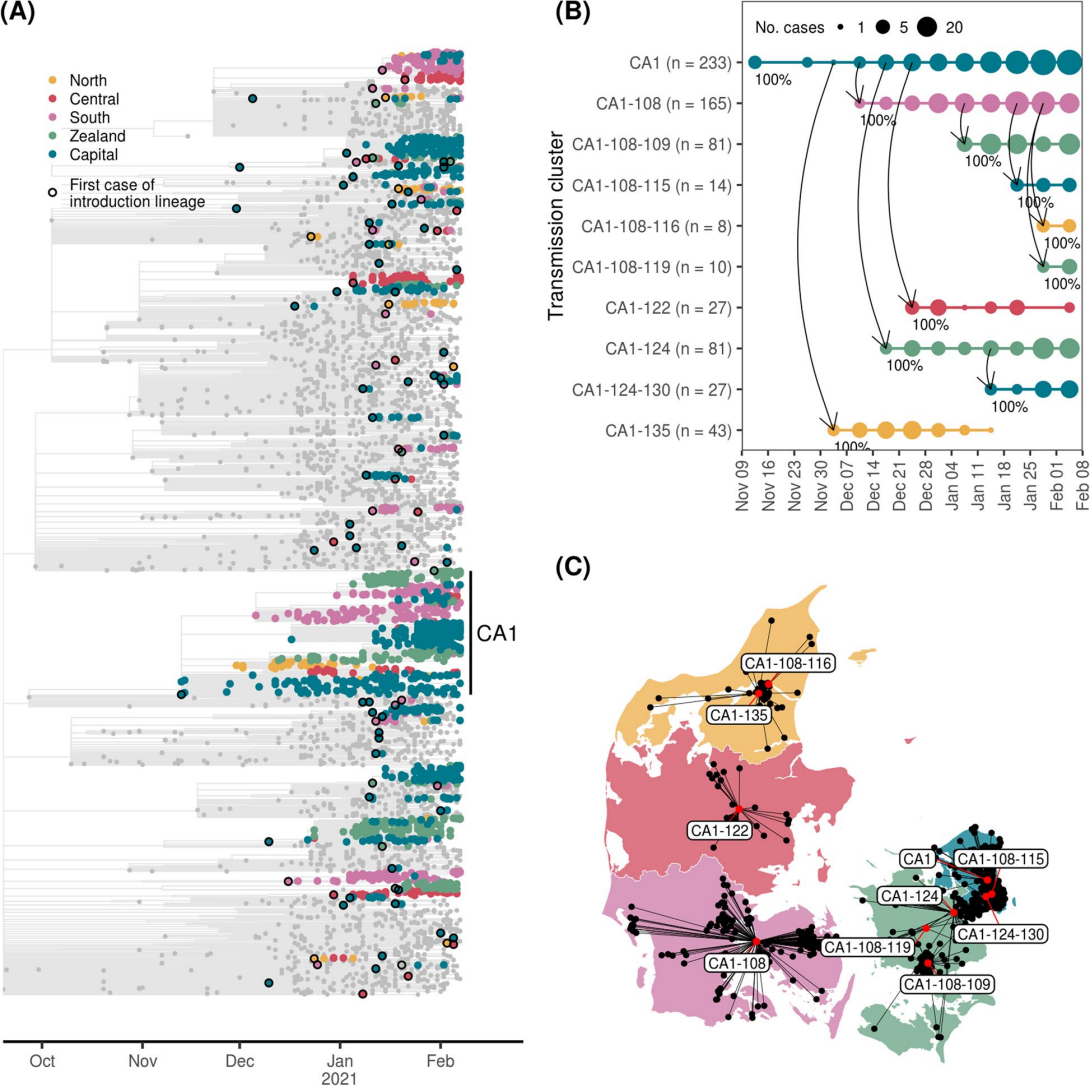
Early introduction of B.1.1.7 and onwards transmission between Danish regions. **A** Time-adjusted phylogenetic tree. Tree branches and non-Danish sequences are colored gray. First sequence in a transmission lineage is used to infer time of importation and highlighted with a black circle. **B**, **C** A focused temporal analysis of the CA1 introduction lineage, focusing on descending clusters with more than 5 cases. **B** The temporal span and dynamics of onwards transmission across regions. Each descendent transmission cluster of CA1 is indicated with a solid horizontal line, with points at each week sized according to the number of cases. The vertical curved arrows indicate direction of transmission. The marginal probability of the observed transmission link is indicated at each arrow. In **C**, the corresponding cases are placed on a map of Denmark using black points, with up to 5 km of random jitter added. Cases from the same transmission cluster are linked by black lines to their centroid location indicated by a red dot. Labels indicate cluster IDs

**Fig. 3:**
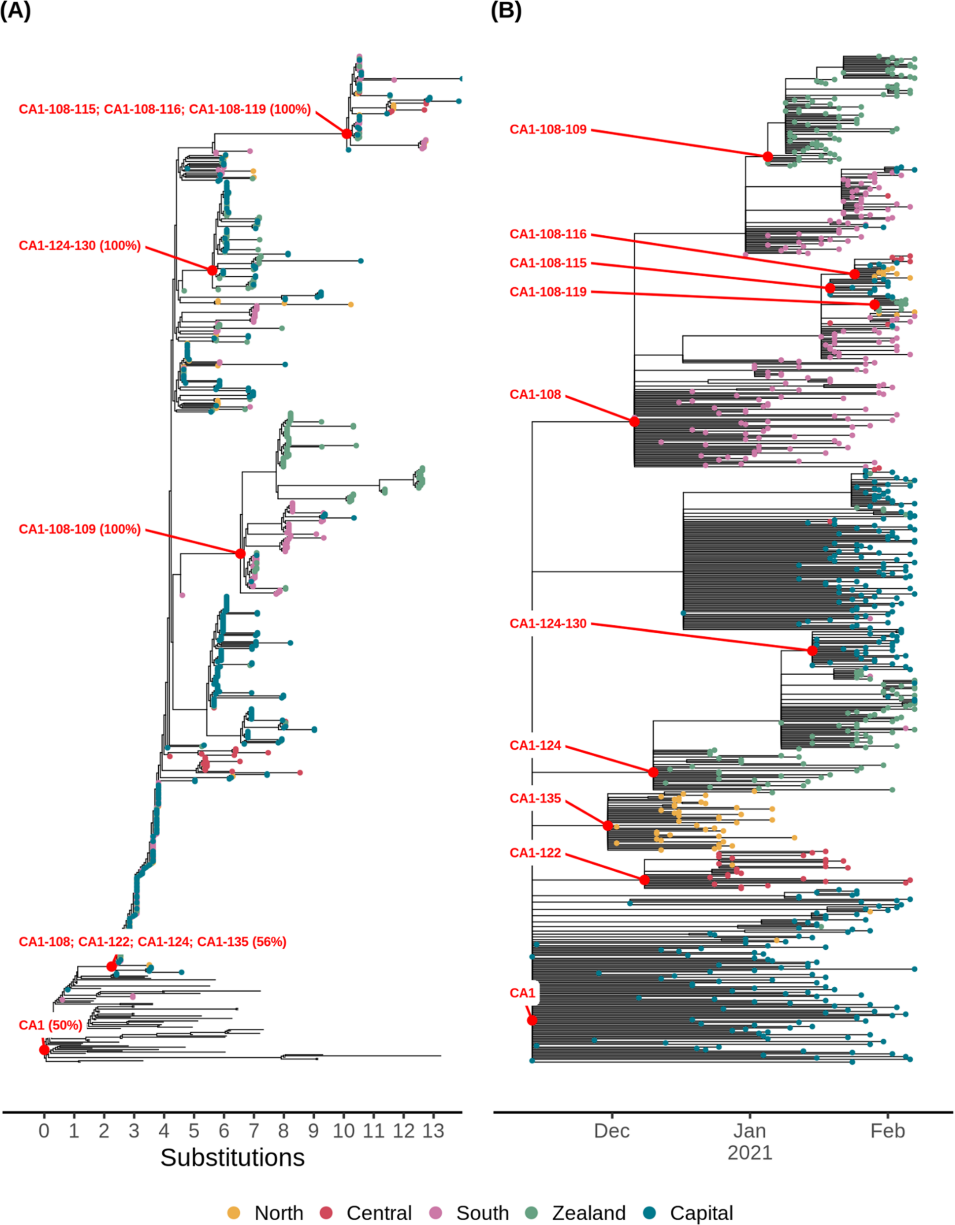
The phylogenetic structure of the data behind the introduction lineage CA1. The data for introduction lineage CA1 and descendant transmission clusters as shown in Fig. 2B. Each point is colored according to region, branches without points are sequences from outside Denmark. Red dots indicate the root node of each cluster with labels. **A** The maximum likelihood tree with branch support indicated as percentage values generated from 1000 ultrafast-bootstrap iterations using the -B option in IQ-tree. Polytomy nodes are collapsed by “;” in the labels. **B** Time-scaled, pruned version of the tree in **A** used for ancestral state reconstruction

**Fig. 4:**
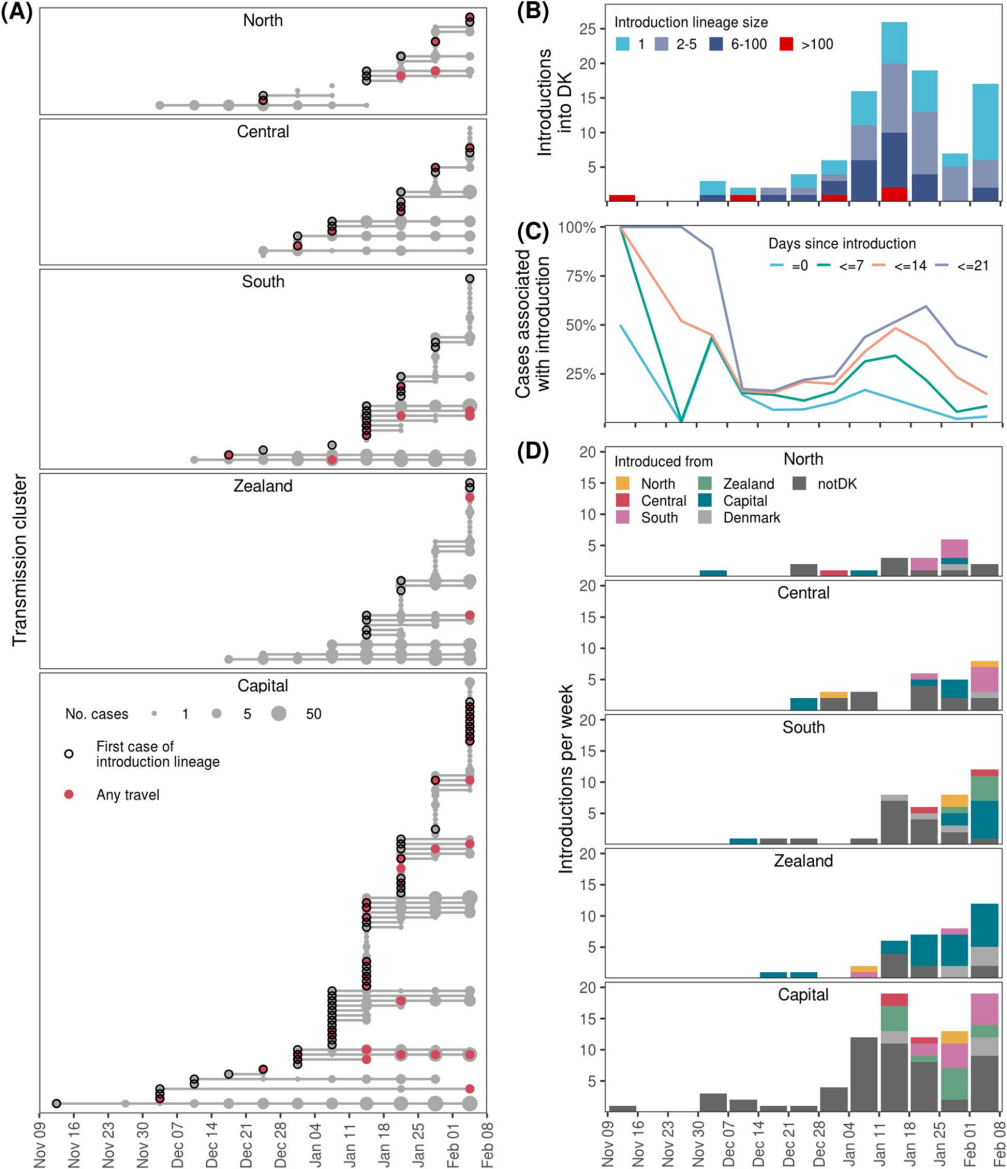
Analysis of importation events into Denmark and onwards transmission between regions. **A** The temporal span and development of each transmission cluster, grouped by Danish region. Each transmission cluster is indicated with a solid horizontal line, with points at each week sized according to the number of cases. Circles indicate first observed case of an introduction lineage imported from outside Denmark. Red points indicate presence of cases with travel history in the given week. **B** The number of introductions into Denmark across time. Colors indicate the total number of offspring cases associated with each introduction lineage. **C** The relative contribution of introductions versus ongoing existing transmission. Introduction-related cases were defined with various cutoffs for the maximum number of days between the case and the first observed case for each introduction lineage, as indicated by different colors. **D** The origin of introductions across time for each region. If there was equal support for multiple regions as origin for an introduction Denmark was used as the origin. Label notDK indicates an introduction from outside Denmark. Only ancestral state changes with a marginal probability > 95% were included in the analysis

Introductions into Denmark and onward viral migration between regions were investigated using a time-scaled phylogenetic tree of 1976 Danish B.1.1.7 genomes and 3611 representative international sequences from outside Denmark (Fig. 2A). We used pastML [39] to perform an ancestral state reconstruction, to infer if sequences were introduced from outside Denmark or transmitted from another Danish region. We define an introduction lineage as a phylogenetic group of Danish sequences that share the same introduction event, similar to the definition of UK transmission lineages in [16]. We do not infer the timing of introduction events from the phylogeny [9, 16, 44], but instead use the first occurrence of an introduction lineage as a proxy justified by the intense testing and high sequencing rate (Additional file 1: Fig. S1). An introduction lineage can be further subdivided into transmission clusters from the ancestral state reconstruction, which is based on the regional and temporal sub-distributions of an introduction lineage. The inferred connections between transmission clusters outline the direction of viral migration between regions. The first cases of B.1.1.7 observed in the Capital region of Denmark on 14 November 2020 defines the introduction lineage CA1, which contained the majority of Danish sequences (736 of 1976, 37%) across all Danish regions (Fig. 2B). The maximum likelihood tree and corresponding time-scaled tree is shown for the CA1 introduction lineage in Fig. 3. We tried to resolve the origin of CA1, but detailed epidemiological investigations revealed no direct or indirect travel link and the ancestral state could not be resolved with good confidence. The CA1 introduction lineage is associated with the first reported cases of B.1.1.7 in all Danish regions, including the first North Denmark transmission cluster CA1-135. This transmission cluster was part of a large but local outbreak verified by epidemiological investigations and primarily associated with a single local superspreading event (haplotype H77, Additional file 1: Fig. S2B). CA1-135 dominated the North Denmark cases until it was last observed in week 2 (Fig. 2B). This contrasts the course of the first transmission clusters in other regions, which showed similar initial growth (CA1-108 in South Denmark and CA1-124 in Zealand) but remained at high prevalence throughout the study period. Interestingly, all transmission clusters appearing up until week 1 in North Denmark were not observed in the remaining study period, which were not true for other regions where a constant level of sustained transmission was present for many larger transmission clusters (Fig. 4A). In the North region, 7 of 11 municipalities had elevated restrictive measures until 16 November 2020 due to circulation of mink-related lineages [42]. The intensified contact-tracing and genomic surveillance in that period likely kept the reproductive numbers for North Denmark below that of other Danish regions, making it difficult for any cluster to develop from local to sustained transmission. As the transition to sustained transmission likely happened during the modeling period, this could also explain why the model underestimated expansion of B.1.1.7 in the North region after the study period ended (Additional file 1: Fig. S2A). Case numbers of B.1.1.7 in South Denmark and Capital also increased to higher levels than what was predicted by our model (Additional file 1: Fig. S2A), likely caused by large superspreading events, evident from the rapid increase of unique local haplotypes in South (H17 and H87) and Capital (H43) regions (Additional file 1: Fig. S2B).

Overall, we identified 103 introductions and 107 viral migration events between regions of B.1.1.7 in the study period (Fig. 4). The majority of introductions occurred during the first half of January 2021 (Fig. 4B); 37 introductions showed no onward transmission, 36 introductions had between 2 and 5 cases of whom 8 exclusively had transmission within the same household, and only 5 caused > 100 cases. To investigate the impact of introductions across time, we considered early cases of the same introduction lineage to be introduction-related, while later cases occur due to sustained transmission in society. To evaluate this, we increment the number of follow-up days for cases to be assigned as introduction-related (Fig. 4C). Overall, the relative contribution of introduction-related cases peaked during onset of B.1.1.7 and again in mid-January (Fig. 4C). Introductions alone (follow-up =0 days in Fig. 4C) accounted for around 5–10% of all cases with substantial temporal variation. Using up to 14 days follow-up since the first case of an introduction to define introduction-related cases within the same introduction lineage, there were periods where > 50% of cases were due to importation from outside Denmark (Fig. 4C).

In collaboration with the Danish Patient Safety Authority [22] which administers the contact tracing efforts in Denmark, we performed detailed epidemiological investigations of registry data to identify direct or indirect travel links for all 1976 cases in this study. A total of 62 persons infected with B.1.1.7 (3%) had recorded travel history to 23 different destinations. The most frequent destinations were the United Arab Emirates (*n* = 21), United Kingdom (*n* = 5), and Pakistan (*n* = 4). We were able to associate 32 (31%) phylogenetically inferred introductions directly to traveling (Fig. 4A, Additional file 1: Fig. S3). We performed a separate ancestral state reconstruction with country-level resolution on international sequences, allowing us to compare the phylogenetically inferred country of origin to the travel history. Of 33 introductions with travel history, 9 (27%) matched by country and 12 (36%) by high-level geographical region.

From the ancestral state reconstruction, we inferred the temporal dynamics of B.1.1.7 dispersion between the five major administrative regions of Denmark. The majority of transmission was local and occurred within regions, but would to a large extent cease if not fueled by introductions from other regions or outside Denmark (Fig. 4D). The Capital and South Denmark regions with international borders and substantial international travel-activity also received the most introductions, possibly a combined effect of multiple factors such as leisure travel and commuting for work. A similar conclusion was reached by normalizing according to the population size of each region (Additional file 1: Fig. S4), except for the Zealand region which relative to its population size received a high amount of importations. Interestingly, the Capital region also had the highest number of outgoing viral migration events to other regions, acting as an important source for sustaining B.1.1.7 in Denmark, particularly in the Zealand region which has a large number of residents commuting to the Capital region on a regular basis.

## Discussion

In many countries, the takeover of B.1.1.7 has prolonged or induced further restrictions to sustain epidemic control. In Denmark, genomic surveillance has been key to rapidly predict growth of B.1.1.7, leading to enforced restrictions in effect from 5 January 2021 when B.1.1.7 was still below 2% relative abundance of all SARS-CoV-2 variants [45]. Enforced restrictions practically eliminated all other circulating variants within 2 months (Fig. 1B) and enabled epidemic control to be maintained as society was gradually reopened from 8 February 2021. We estimate an increased transmissibility that is in line with estimates made from previous studies [7–9, 12]. However, it is possible that the rising frequency of B.1.1.7 across Europe during the study period cased a greater number of B.1.1.7 introductions which may have inflated the estimated transmissibility of B.1.1.7. Restrictions aimed specifically towards B.1.1.7 were in place prior to the modeling period, and the relative number of introductions was lower in the period used for modeling compared to before (Fig. 4C). Therefore, we do not expect this to be of major concern, but further studies would be needed to verify this [46].

In this study, we show retrospectively how B.1.1.7 emerged in Denmark and subsequently spread nationwide. Similar studies have investigated the spatiotemporal dynamics during the early stages of B.1.1.7 in the UK [19] and USA [8, 9]. In the UK, evidence suggests that B.1.1.7 emerged from one defined geographical area with substantial within-area transmission before spreading across the country [19]. We found the development of B.1.1.7 in Denmark to be similar, as the initial CA1 introduction lineage had sustained itself within the region for several weeks before spreading to all other Danish regions within 4 weeks (Fig. 2B). Elevation of B.1.1.7 to VOC status occurred on 18 December 2020, few weeks after it was first observed in the Capital region in November 2020. Therefore, CA1 may have developed substantially through undetected transmission within the Capital region. Its high prevalence and transmission could have caused a spill-over effect to other regions, accelerating growth of this lineage in those places. This has been reported in the UK and may explain why estimates of B.1.1.7 transmissibility in the UK dropped from December 2021 onwards, as early estimates were too high [19]. In Denmark, similar declines in transmissibility were observed during January 2021 [47]. Although we did not identify region-specific estimates of transmissibility, we observed inter-regional differences in early B.1.1.7 expansion. Our genomic analysis revealed that the early expansion in the North region could be associated to a single localized cluster which did not cause onward transmission and hence likely delayed B.1.1.7 expansion in the North region by a few weeks (Fig. 1C). These observations and conclusions of the observed dynamics at regional level were reached independently of any detailed demographic data, providing explanatory context to the overall observed epidemiological patterns of prevalence. Importantly, we were able to further verify the validity of these genomic findings using data from authority records, highlighting the power of coupling genomic data with other data sources.

Our study is the first to outline introduction dynamics of B.1.1.7 on a nation-wide level, as comparative studies either did not do it [19] or the dataset was too sparse to attempt such analysis [9]. From 22 December 2020 onwards, authorities enforced elevated restrictions for travelers from the UK to halt B.1.1.7 expansion [21]. This did not, however, affect the proportion of cases derived from introductions, which increased from December 2020 to mid-January 2021 (Fig. 4C). Similar measures were enforced for inbound travelers from Italy and Austria in the spring of 2020 to stop SARS-CoV-2 from spreading to Denmark [48], questioning the efficiency of country-specific restrictions and non-enforced recommendations of self-isolation after travel. However, the extent of this cannot be derived from the data in this study alone and does not necessarily generalize to other countries with different restrictive measures. In addition, we found only limited agreement between reported travel history and that inferred by the ancestral state reconstruction. This highlights the limitations of available genomic data, which is still heavily biased both geographically and temporally [49, 50], making high-resolution inference of introduction origin from phylogenetic data alone very difficult.

In contrast to other relevant studies [9, 16], which uses time of the most recent common ancestor (tMRCA) to time an introduction event, we considered the first observation of an introduction lineage as a proxy for an introduction event. Adequate sampling of the population is crucial to accurately infer timing of introduction events [16]. In this study, between 25 and > 75% of the positive cases were sequenced (Additional file 1: Fig. S1), comparable to frequencies of Australia and Iceland [49]. It is also higher than the average 10% coverage in the UK, one of the countries which numerically have sequenced the most [49]. Therefore, we do not expect lack of sampling to influence the findings of this study.

VOC designation of B.1.1.7 and upscaling of capacity in Denmark was too late to hinder the takeover of this lineage, which had to be managed by broad societal restrictions. Authorities were not able to do sufficient genome-based contact tracing due to low number of samples sequenced (around 25% of positive cases) and slow turnaround (median 11 days). These levels are still among the highest in the world [49] and have repeatedly enabled Danish authorities to perform accurate forecasting and adjust interventions accordingly. Since January 2021 Denmark has gradually improved its genomic surveillance, currently reaching a sequencing capacity of > 90% of positive cases and a median 4 days turnaround. In addition, the introduction of rapid screening assays for hallmark mutations [51, 52] has enabled ultra-fast initiation of contact tracing and outbreak investigations.

We are now at a level in Denmark that enables targeted contact tracing of VOCs like B.1.351 [53] (Beta) and P.1 [54] (Gamma), which have been kept at low prevalence since first observed on 10 January and 26 February 2021, respectively [5]. This has been possible due to limited importation intensity and local outbreaks, making ideal conditions for efficient contact tracing. It was, however, not possible to control B.1.617.2 (Delta) albeit some delays were successful, as it was kept below 1% prevalence for two months before completely displacing B.1.1.7 during June 2021 [5]. B.1.617.2 is more transmissible than B.1.1.7 [55], and after sustained community transmission started to occur in June, it quickly became dominant making B.1.617.2 focused contact tracing meaningless. This is also the reason why all variant-specific contact tracing was suspended from July 5, 2021 [56].

## Conclusions

Collectively, we outline the introduction and transmission of B.1.1.7 in Denmark using high-throughput genomic surveillance and comprehensive register data. We identified an increased transmissibility of B.1.1.7, in line with findings from other studies. In addition, our study is one of only a few describing the spatiotemporal dynamics of B.1.1.7 at a national level [8, 9, 19] and the only study to our knowledge that comparatively assess the impact of foreign introductions vs inter-region viral migration of B.1.1.7. Both types of spread caused onward transmission that constituted a substantial portion of cases, in a period of travel and community restrictions. Maintaining a high level of genomic surveillance in Denmark, despite its currently limited usage for contact tracing, has been pivotal to enable swift response in the face of new VOCs, the origin and timeliness of which are virtually impossible to predict. However, a global coordinated effort of genomic surveillance coupled with epidemiological and contact tracing data is needed to track and mitigate transmission of VOCs [19]. This is perhaps even more important as vaccine rollouts are happening unequally across the globe, posing very different adaptive challenges for SARS-CoV-2, the consequences of which on viral evolution are difficult to predict [57].

## Supplementary Information

**Additional file 1. **List of Danish COVID-19 Genome Consortium members. **Fig. S1.** Sequencing rate relative to total number of covid19 cases per week (A), relative testing effort (B), and percent positive (C) for each Danish region across time. The two vertical dashed lines indicate the beginning and end of study period used to infer B.1.1.7 transmissibility, while the non-shaded area shows the period used for phylogenetic analysis. The time outside the study are shaded in grey. **Fig. S2.** The two vertical dashed lines indicate the beginning and end of study period used to infer B.1.1.7 transmissibility, while the non-shaded area shows the period used for phylogenetic analysis. The time outside the study are shaded in grey. (A) Model predictions from Poisson regression model on daily counts of B.1.1.7 for each region. Dark-grey areas represent 95% CI. (B) Frequency of unique haplotypes across time for each region. Each line represents the weekly count of a unique B.1.1.7 haplotype. The four haplotypes mentioned in the main text are highlighted. **Fig. S3.** Assessing robustness of inferred introductions from phylogenetic analysis using travel history. (A) and (B) are grouped into introduction lineages that are introduced from abroad and transmission clusters introduced from other Danish regions. (A) shows the number of introduction lineages and transmission clusters with a minimum duration given on the x-axis. (B) shows the percent of introduction lineages and transmission clusters with travel-associated cases before a cutoff day indicated on the x-axis. The cutoff day on x-axis is relative to the first occurrence of the introduction lineage or transmission cluster. **Fig. S4**. Alternative version of Fig. 4D, showing the origin of introductions across time for each region. The y-axis is scaled to introductions per week per 100,000 inhabitants based on the population size for each region. If there was equal support for multiple regions as origin for an introduction Denmark was used as the origin. Label notDK indicates an introduction from outside Denmark. Only ancestral state changes with a marginal probability >95% were included in the analysis. **Table S1.** Nucleotide mutations for each of the four haplotypes specifically mentioned in the manuscript.

**Additional file 2.** GISAID accession numbers and acknowledgement table for sequences used in this study.

## Data Availability

SARS-CoV-2 consensus genome sequences associated with this work have been uploaded to the GISAID database (https://www.gisaid.org/) in accordance with Danish law (no. 285 vers. 2021-02-27). Consequently, dates are binned by week and maximum spatial resolution is at regional level. Accession numbers are available in the Additional file 2. All code used to run the phylogenetic analysis, Poisson regression models, and generate the visualizations used in this work is available on github, https://github.com/TYMichaelsen/B117-DK-introduction [57].
