# Supplementary material for: Introduction and transmission of SARS-CoV-2 lineage B.1.1.7, Alpha variant, in Denmark
Source: Genome Med. 2022 May 4;14:47. doi: 10.1186/s13073-022-01045-7 (PMC9064402; doi:10.1186/s13073-022-01045-7)
Supplement: Supplementary file 1 — Additional file 1. List of Danish COVID-19 Genome Consortium members. Fig. S1. Sequencing rate relative to total number of covid19 cases per week (A), relative testing effort (B), and percent positive (C) for each Danish region across time. The two vertical dashed lines indicate the beginning and end of study period used to infer B.1.1.7 transmissibility, while the non-shaded area shows the period used for phylogenetic analysis. The time outside the study are shaded in grey. Fig. S2. The two vertical dashed lines indicate the beginning and end of study period used to infer B.1.1.7 transmissibility, while the non-shaded area shows the period used for phylogenetic analysis. The time outside the study are shaded in grey. (A) Model predictions from Poisson regression model on daily counts of B.1.1.7 for each region. Dark-grey areas represent 95% CI. (B) Frequency of unique haplotypes across time for each region. Each line represents the weekly count of a unique B.1.1.7 haplotype. The four haplotypes mentioned in the main text are highlighted. Fig. S3. Assessing robustness of inferred introductions from phylogenetic analysis using travel history. (A) and (B) are grouped into introduction lineages that are introduced from abroad and transmission clusters introduced from other Danish regions. (A) shows the number of introduction lineages and transmission clusters with a minimum duration given on the x-axis. (B) shows the percent of introduction lineages and transmission clusters with travel-associated cases before a cutoff day indicated on the x-axis. The cutoff day on x-axis is relative to the first occurrence of the introduction lineage or transmission cluster. Fig. S4. Alternative version of Fig. 4D, showing the origin of introductions across time for each region. The y-axis is scaled to introductions per week per 100,000 inhabitants based on the population size for each region. If there was equal support for multiple regions as origin for an introduction Denmark was us [file 13073_2022_1045_MOESM1_ESM.docx]

Supplementary Materials

**Introduction and transmission of SARS-CoV-2 B.1.1.7 (Alpha) in Denmark**

Thomas Y. Michaelsen^1^, Marc Bennedbæk^2^, Lasse E. Christiansen^3^, Mia Sarah Fischer Jørgensen^4^, Camilla Holten Møller^5^, Emil A. Sørensen^1^, Simon Knutsson^1^, Jakob Brandt^1^, Thomas B. N. Jensen^1^, Clarisse Chiche-Lapierre^1^, Emilio F. Collados^1^, Trine Sørensen^1^, Celine Petersen^1^, Vang Le-Quy^6^, Mantas Sereika^1^, Frederik T. Hansen^1^, Morten Rasmussen^7^, Jannik Fonager^7^, Søren M. Karst^7^, Rasmus L. Marvig^8^, Marc Stegger^9^, Raphael N. Sieber^9^, Robert Skov^5^, Rebecca Legarth^4^, Tyra Grove Krause^5^, Anders Fomsgaard^7^, The Danish Covid-19 Genome Consortium (DCGC)^10^, Mads Albertsen^1,*^

Correspondence to: [ma@bio.aau.dk](mailto:ma@bio.aau.dk)

**List of Danish Covid-19 Genome Consortium members**

The ordering of affiliations and authors does not reflect any difference in contribution.

Kasper S. Andersen^1^, Martin H. Andersen^1^, Amalie Berg^1^, Susanne R. Bielidt^1^, Sebastian M. Dall^1^, Erika Dvarionaite^1^, Susan H. Hansen^1^, Vibeke R. Jørgensen^1^, Rasmus H. Kirkegaard^1^, Wagma Saei^1^, Trine B. Nicolajsen^1^, Stine K. Østergaard^1^, Rasmus F. Brøndum^2^, Martin Bøgsted^2^, Katja Hose^3^, Tomer Sagi^3^, Miroslaw Pakanec^3^, David Fuglsang-Damgaard^4^, Mette Mølvadgaard^4^, Henrik Krarup^5^, Christina W. Svarrer^6^, Mette T. Christiansen^6^, Anna C. Ingham^6^, Thor B. Johannesen^6^, Martín Basterrechea^6^, Berit Lilje^6^, Kirsten Ellegaard^6^, Povilas Matusevicius^6^, Lars B. Christoffersen^6^, Man-Hung E. Tang^6^, Kim L. Ng^6^, Sofie M. Edslev^6^, Sharmin Baig^6^, Ole H. Larsen^7^, Kristian A. Skipper^7^, Søren Vang^7^, Kurt J. Handberg^8^, Marc T. K. Nielsen^8^, Carl M. Kobel^8^, Camilla Andersen^8^, Irene H. Tarpgaard^8^, Svend Ellermann-Eriksen^8^, José A. S. Castruita^9^, Uffe V. Schneider^9^, Nana G. Jacobsen^9^, Christian Ø. Andersen^9^, Martin S. Pedersen^10^, Kristian Schønning^10^, Nikolai Kirkby^10^, Lene Nielsen^11^, Line L. Nilsson^11^, Martin B. Friis^11^, Thomas Sundelin^11^, Thomas A. Hansen^11^, Marianne N. Skov^12^, Thomas V. Sydenham^12^, Xiaohui C. Nielsen^13^, Christian H. Schouw^13^, Anders Jensen^14^, Ea S. Marmolin^14^, John E. Coia^15^, Dorte T. Andersen^15^

^1^ Department of Chemistry and Bioscience, Aalborg University.

^2^ Department of Clinical Medicine, Aalborg University.

^3^ Department of Computer Science, Aalborg University.

^4^ Department of Clinical Microbiology, Aalborg University Hospital.

^5^ Department of Molecular Diagnostics, Aalborg University Hospital.

^6^ Statens Serum Institut.

^7^ Department of Molecular Medicine (MOMA), Aarhus University Hospital.

^8^ Department of Clinical Microbiology, Aarhus University Hospital.

^9^ Department of Clinical Microbiology, Copenhagen University Hospital.

^10^ Rigshospitalet, Copenhagen University Hospital.

^11^ Department of Clinical Microbiology, Herlev Hospital.

^12^ Odense University Hospital.

^13^ Zealand University Hospital.

^14^ Sygehus Lillebælt.

^15^ Department of Clinical Microbiology, Sydvestjysk sygehus.

**
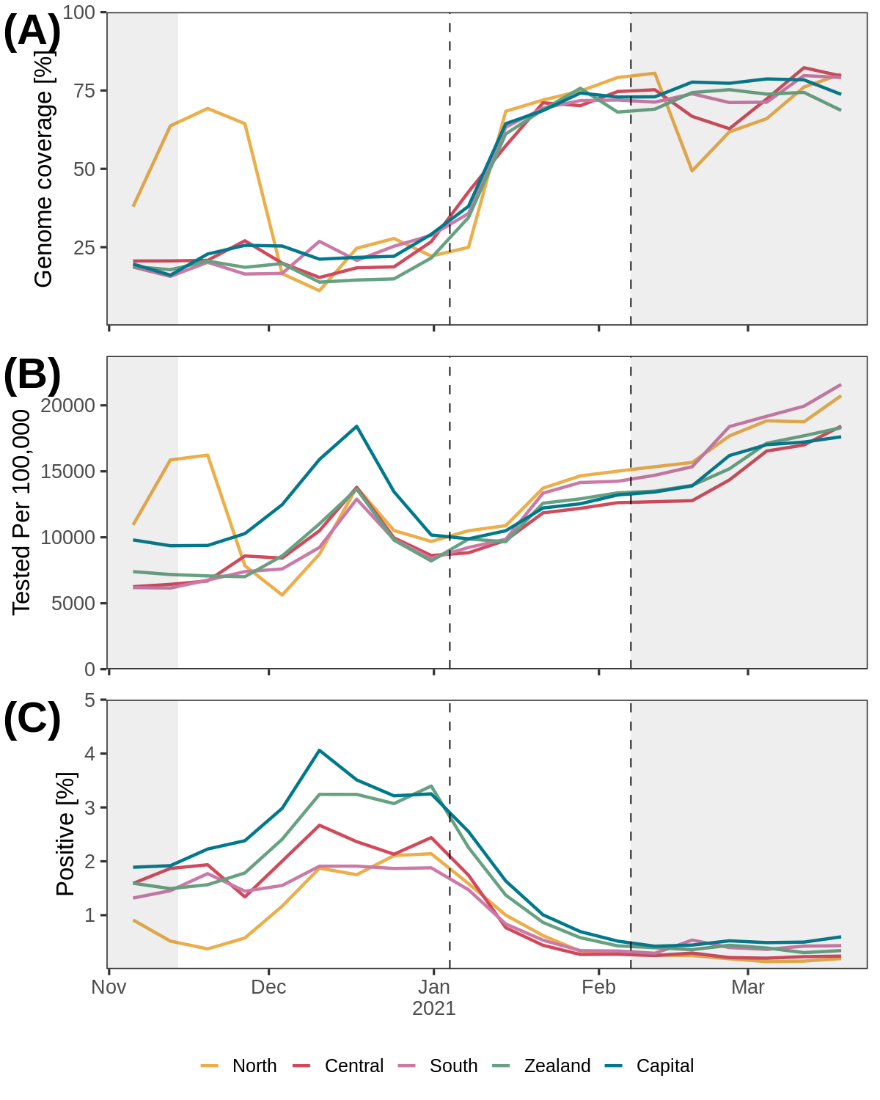
**

**Fig. S1.** Sequencing rate relative to total number of covid19 cases per week **(A)**, relative testing effort **(B)**, and percent positive **(C)** for each Danish region across time. The two vertical dashed lines indicate the beginning and end of study period used to infer B.1.1.7 transmissibility, while the non-shaded area shows the period used for phylogenetic analysis. The time outside the study are shaded in grey.


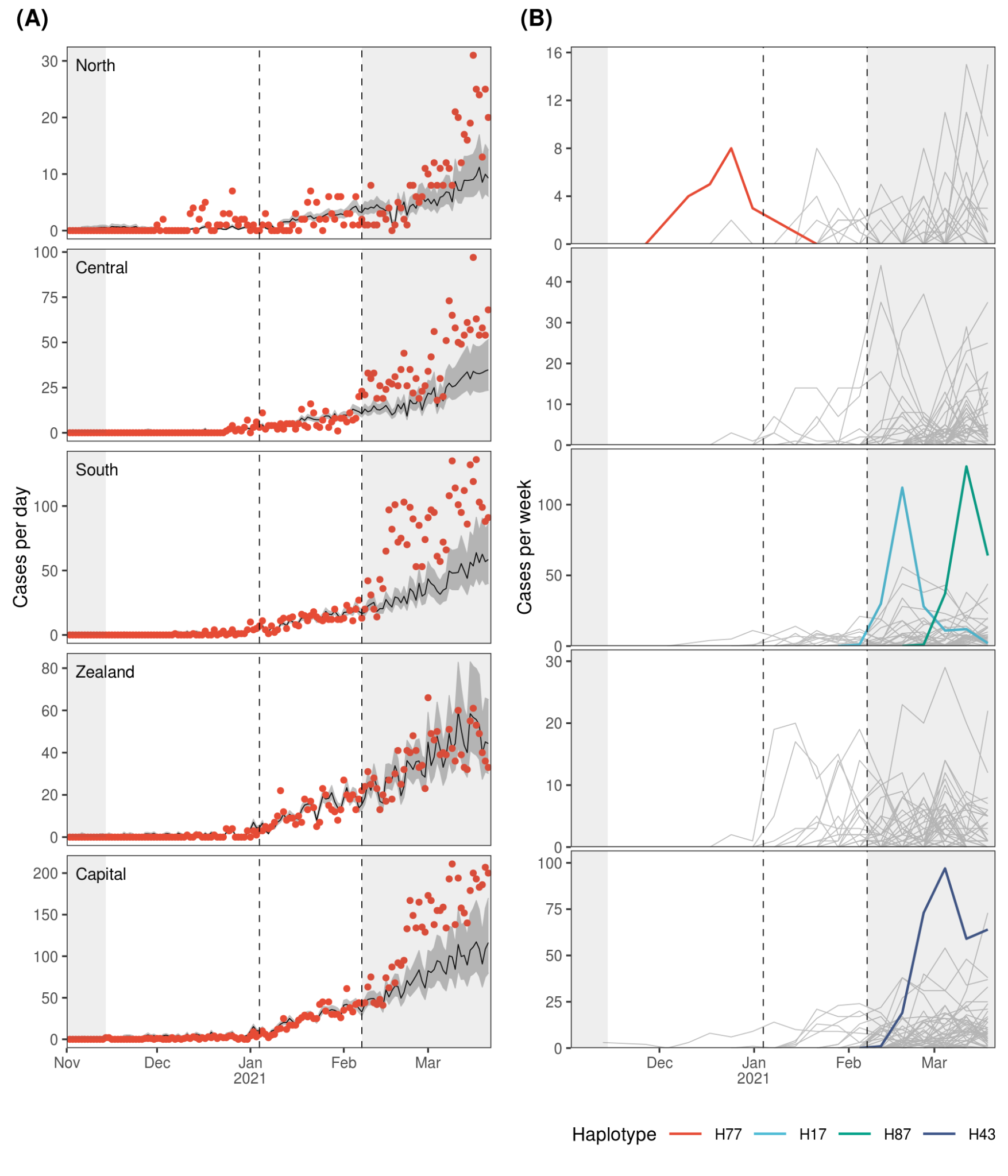


**Fig. S2.** The two vertical dashed lines indicate the beginning and end of study period used to infer B.1.1.7 transmissibility, while the non-shaded area shows the period used for phylogenetic analysis. The time outside the study are shaded in grey. (**A**) Model predictions from Poisson regression model on daily counts of B.1.1.7 for each region. Dark-grey areas represent 95% CI. (**B**) Frequency of unique haplotypes across time for each region. Each line represents the weekly count of a unique B.1.1.7 haplotype. The four haplotypes mentioned in the main text are highlighted.

**
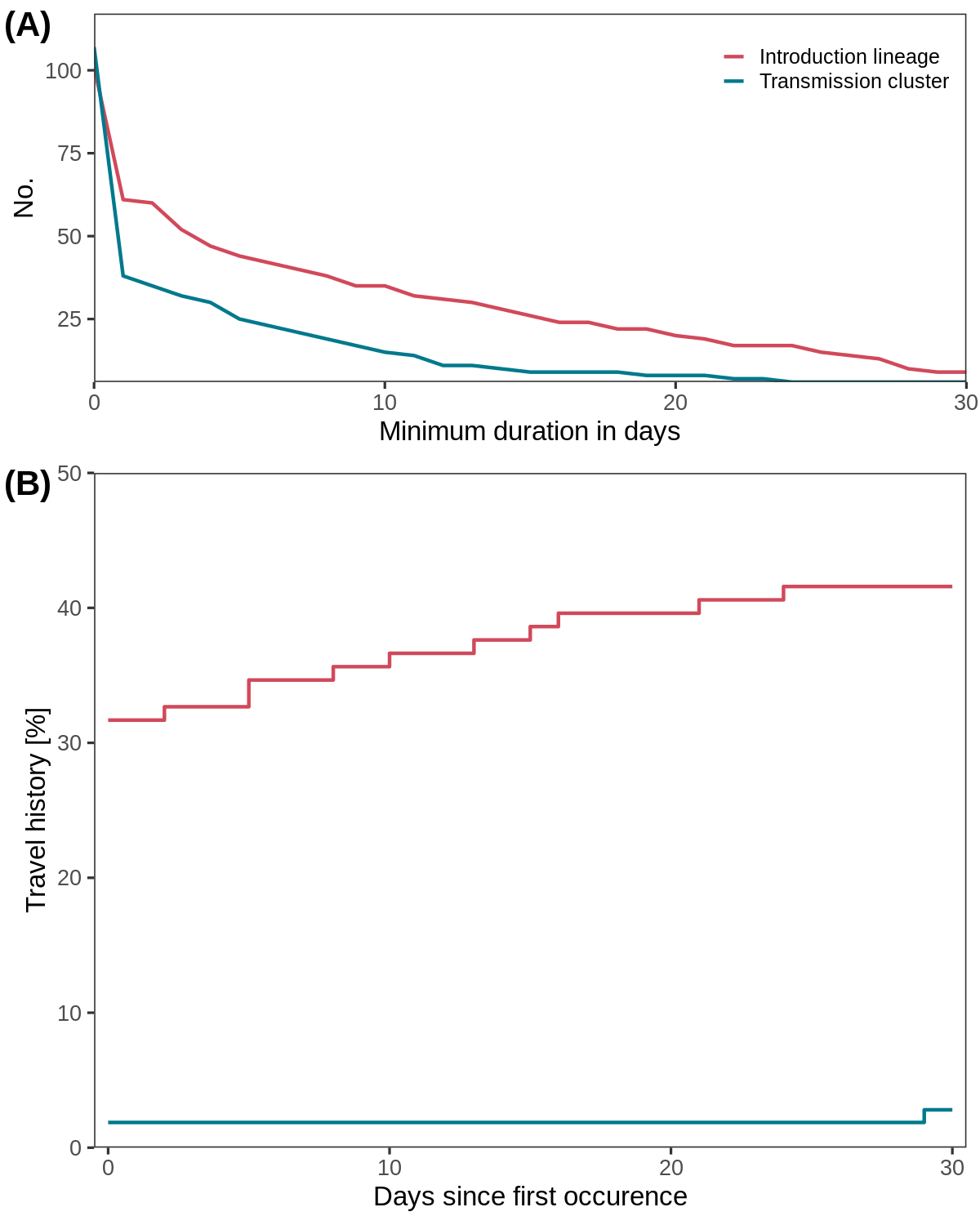
**

**Fig. S3.** Assessing robustness of inferred introductions from phylogenetic analysis using travel history. (**A**) and (**B**) are grouped into introduction lineages that are introduced from abroad and transmission clusters introduced from other Danish regions. (**A**) shows the number of introduction lineages and transmission clusters with a minimum duration given on the x-axis. (**B**) shows the percent of introduction lineages and transmission clusters with travel-associated cases before a cutoff day indicated on the x-axis. The cutoff day on x-axis is relative to the first occurrence of the introduction lineage or transmission cluster.


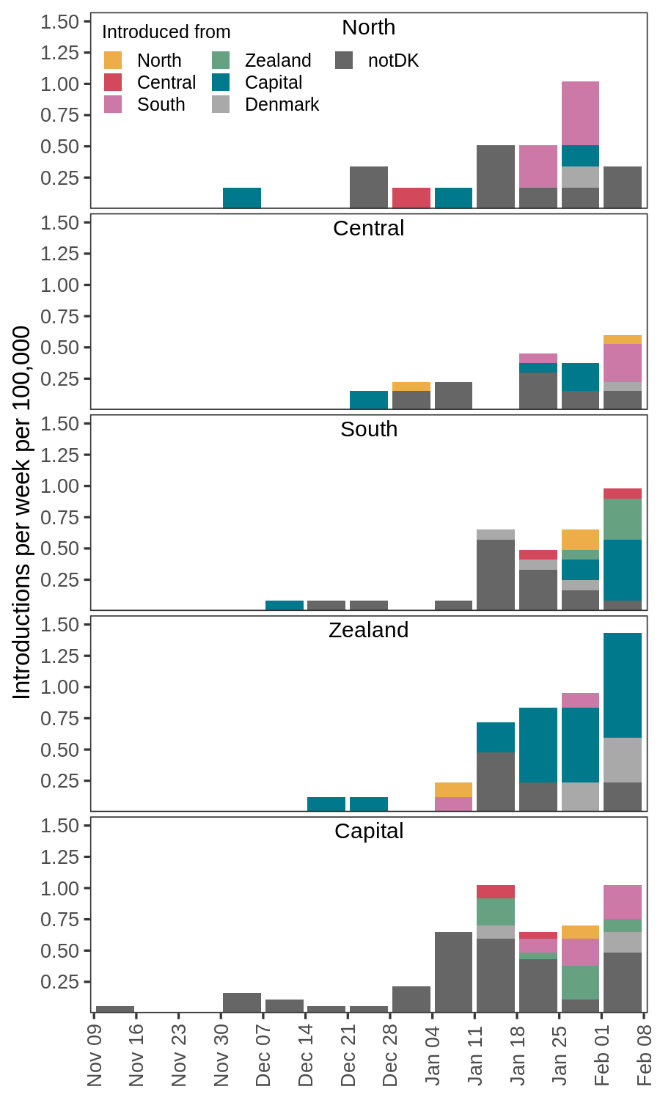


**Fig. S4**. Alternative version of Fig. 4D, showing the origin of introductions across time for each region. The y-axis is scaled to introductions per week per 100,000 inhabitants based on the population size for each region. If there was equal support for multiple regions as origin for an introduction Denmark was used as the origin. Label notDK indicates an introduction from outside Denmark. Only ancestral state changes with a marginal probability >95% were included in the analysis.

| **Haplotype** | **Nucleotide mutations** |
| --- | --- |
| H43 | C241T, C913T, C3037T, C3267T, C5388A, C5986T, T6954C, C14408T, C14676T, T15096C, C15279T, T16176C, A17496G, T22016A, A23063T, C23271A, A23403G, C23604A, C23709T, T24506G, G24914C, G25785T, C27972T, G28048T, A28095T, A28111G, G28280C, A28281T, T28282A, G28881A, G28882A, G28883C, C28977T, G29764A, T11288-, C11289-, T11290-, G11291-, G11292-, T11293-, T11294-, T11295-, T11296-, A11297-, A21766-, C21767-, A21768-, T21769-, G21770-, T21771-, C21772-, T21994-, T21995-, A21996-, C21997-, A28274- |
| H77 | C241T, C913T, C2110T, C3037T, C3177T, C3267T, C5388A, C5986T, T6954C, C14408T, C14676T, C15279T, T16176C, A23063T, C23271A, A23403G, C23604A, C23709T, T24506G, G24914C, C27972T, G28048T, A28111G, G28280C, A28281T, T28282A, G28881A, G28882A, G28883C, C28977T, T11288-, C11289-, T11290-, G11291-, G11292-, T11293-, T11294-, T11295-, T11296-, A11297-, A21766-, C21767-, A21768-, T21769-, G21770-, T21771-, C21772-, T21994-, T21995-, A21996-, C21997-, A28274- |
| H87 | C241T, C913T, C2623T, C3037T, C3267T, C4009T, C5388A, T5762C, C5986T, T6954C, C14408T, C14676T, G15025T, C15279T, T16176C, A17615G, T19050C, A23063T, C23271A, A23403G, C23604A, C23638T, C23709T, T24506G, G24914C, C27972T, G28048T, A28111G, G28280C, A28281T, T28282A, G28881A, G28882A, G28883C, C28977T, T11288-, C11289-, T11290-, G11291-, G11292-, T11293-, T11294-, T11295-, T11296-, A11297-, A21766-, C21767-, A21768-, T21769-, G21770-, T21771-, C21772-, T21994-, T21995-, A21996-, C21997-, A28274- |
| H17 | C241T, C913T, C3037T, C3267T, C5388A, C5944T, C5986T, T6954C, A10716G, C14408T, C14676T, T15096C, C15279T, T16176C, A23063T, C23271A, A23403G, C23604A, C23709T, C23854T, T24506G, G24914C, G26951C, C27972T, G28048T, A28095T, A28111G, G28280C, A28281T, T28282A, G28881A, G28882A, G28883C, G28884C, C28977T, T11288-, C11289-, T11290-, G11291-, G11292-, T11293-, T11294-, T11295-, T11296-, A11297-, A21766-, C21767-, A21768-, T21769-, G21770-, T21771-, C21772-, T21994-, T21995-, A21996-, C21997-, A28274- |

**Table S1.** Nucleotide mutations for each of the four haplotypes specifically mentioned in the manuscript.
